# Pelvic Ring Fractures: A Biomechanical Comparison of Sacral and Lumbopelvic Fixation Techniques

**DOI:** 10.3390/bioengineering11040348

**Published:** 2024-04-02

**Authors:** Sudharshan Tripathi, Norihiro Nishida, Sophia Soehnlen, Amey Kelkar, Yogesh Kumaran, Toshihiro Seki, Takashi Sakai, Vijay K. Goel

**Affiliations:** 1 Engineering Center for Orthopedic Research (E-CORE), Department of Bioengineering and Orthopaedic Surgery, University of Toledo, Toledo, OH 43606, USA; sudharshan.tripathi@rockets.utoledo.edu (S.T.); sophia.soehnlen@rockets.utoledo.edu (S.S.); amey.kelkar@rockets.utoledo.edu (A.K.); yogesh.kumaran@rockets.utoledo.edu (Y.K.); 2 Department of Orthopedic Surgery, Yamaguchi University Graduate School of Medicine, 1-1-1 Minami-Kogushi, Ube 755-8505, Yamaguchi, Yamaguchi Prefecture, Japan; nishida3@yamaguchi-u.ac.jp (N.N.); nsd38156@gmail.com (T.S.); cozy@yamaguchi-u.ac.jp (T.S.)

**Keywords:** pelvic ring fractures, fracture displacement, sacroiliac joint, fixation techniques, finite element analysis

## Abstract

Background Context: Pelvic ring fractures are becoming more common in the aging population and can prove to be fatal, having mortality rates between 10% and 16%. Stabilization of these fractures is challenging and often require immediate internal fixation. Therefore, it is necessary to have a biomechanical understanding of the different fixation techniques for pelvic ring fractures. Methods: A previously validated three-dimensional finite element model of the lumbar spine, pelvis, and femur was used for this study. A unilateral pelvic ring fracture was simulated by resecting the left side of the sacrum and pelvis. Five different fixation techniques were used to stabilize the fracture. A compressive follower load and pure moment was applied to compare different biomechanical parameters including range of motion (contralateral sacroiliac joint, L1-S1 segment, L5-S1 segment), and stresses (L5-S1 nucleus stresses, instrument stresses) between different fixation techniques. Results: Trans-iliac–trans-sacral screw fixation at S1 and S2 showed the highest stabilization for horizontal and vertical displacement at the sacral fracture site and reduction of contralateral sacroiliac joint for bending and flexion range of motion by 165% and 121%, respectively. DTSF (Double transiliac rod and screw fixation) model showed highest stabilization in horizontal displacement at the pubic rami fracture site, while the L5_PF_W_CC (L5-Ilium posterior screw fixation with cross connectors) and L5_PF_WO_CC (L5-Ilium posterior screw fixation without cross connectors) showed higher rod stresses, reduced L1-S1 (approximately 28%), and L5-S1 (approximately 90%) range of motion. Conclusions: Longer sacral screw fixations were superior in stabilizing sacral and contralateral sacroiliac joint range of motion. Lumbopelvic fixations displayed a higher degree of stabilization in the horizontal displacement compared to vertical displacement of pubic rami fracture, while also indicating the highest rod stresses. When determining the surgical approach for pelvic ring fractures, patient-specific factors should be accounted for to weigh the advantages and disadvantages for each technique.

## 1. Introduction

The mechanical integrity of the pelvic ring is attributable to the pelvis’ highly stable structure [1]. However, due to the aging population, fragility fractures are becoming more common. These fractures can be the result of low energy impact, or high-velocity injuries such as motor vehicle accidents, falls from large heights, and crush injuries [2]. Pelvic ring injuries can prove to be fatal and disrupt the associated vascular and neurologic structures, especially in geriatric patients as mortality rates range between 10% and 16% [3]. Classification of fractures involve many groups, including the Rommen classification for fragility fractures of the pelvic ring. In the Rommen classification, the type IIC fracture is a variant which is a non-displaced sacral, iliosacral, or ilium fracture with anterior disruption [2]. The complete disruption of the posterior and anterior pelvic complex in these fractures posit a highly demanding injury and unilateral pelvic instability [3,4]. Type IIC pelvic fractures are challenging in that they often require immediate stabilization which is typically performed through internal fixation to maintain circulatory balance and to achieve anatomic restoration of the pelvis [5,6,7].

Several pelvic ring fracture fixation techniques currently exist with varying levels of invasiveness. Transiliac plate fixation, which connects the left and right ilium with plates, is highly invasive to posterior soft tissues of the pelvis. To mitigate this, recent literature has investigated several minimally invasive treatment approaches that have been found to be equally stable [5,8]. These treatments include the trans-iliac–trans-sacral screw (TITS) [9], the ilio-sacral (IS) screw [10,11], the transiliac rod and screw fixation (TIF) procedure and lumbopelvic fixation (LP) [8,12,13,14]. Several biomechanical studies have been conducted to assess the fixation techniques for fracture treatment. Acklin et al. [15] and Hu et al. [16] compared IS fixation with lumbopelvic fixations for the treatment of Denis Fractures and concluded that lumbopelvic fixations are biomechanically stable; however, such fixation compromises the mobility of the lumbar region. These studies primarily focused on comparing the results of the range of motion at the sacroiliac joint without information about the displacement around the fracture area. Zhao et al. [17] compared 2 IS and 2 TITS configuration for treating unilateral sacral fractures and concluded that 2 IS fixations are more biomechanically stable. However, regarding pelvic ring fracture, no previous biomechanical in silico study examined the displacement at the fracture site for sacral and lumbopelvic fixation techniques. Therefore, the current FE study aimed to evaluate the vertical and horizontal displacement at the fracture area between sacral and lumbopelvic fixation techniques. By utilizing a three-dimensional finite element (FE) model, this biomechanical study compares different combinations of five minimally invasive treatments for unstable type IIC pelvic fractures.

## 2. Material and Methods

### 2.1. Finite Element Model of Lumbar Spine Pelvis

**Figure 1:**
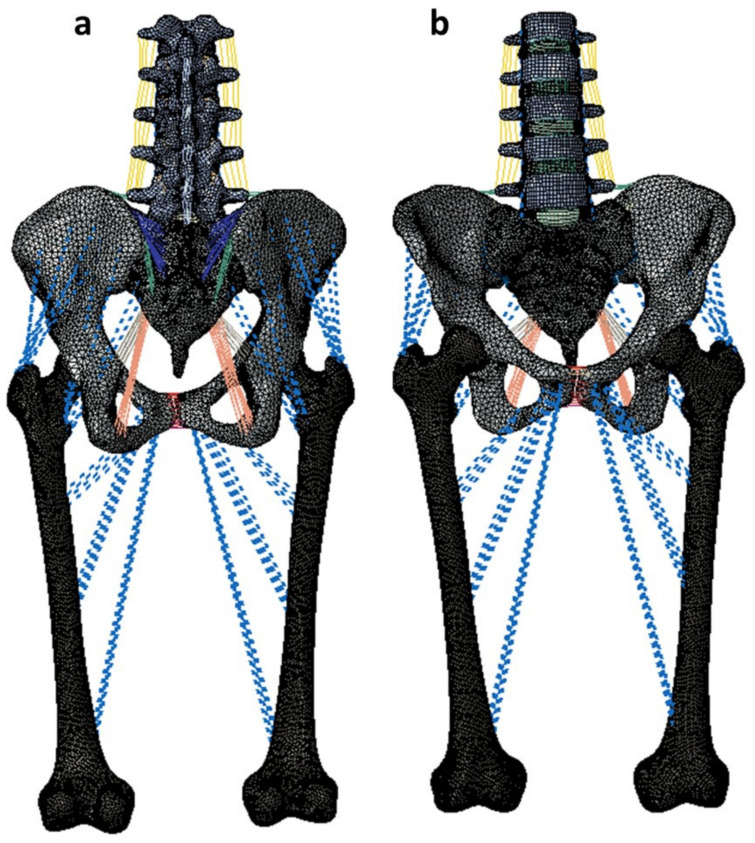
Intact female model developed from CT images of a female with no abnormalities or degenerative changes. (**a**) indicates the posterior view while (**b**) indicates the anterior view.

A non-linear ligamentous FE model including the lumbar spine, sacrum, pelvis, and femurs was used for analyses using Abaqus 2019 (Dassault Systèmes, Simulia Inc., Providence, RI, USA) (Figure 1). The FE model was developed from the CT scan images of a female cadaveric spine without any abnormalities, deformities, or severe degenerative changes. The CT images were imported to Mimics (Materialize Inc., Leuven, Belgium) for model reconstruction.

The FE model included the intervertebral discs, ligamentous structures, vertebral bodies, and facet joints. The initial lumbar lordosis (LL) for the adult female model was 42° representing normal spinal alignment as per Schwab classification [18]. The other spinopelvic parameters of this model were sacral slope (SS) = 26°, pelvic incidence (PI) = 37°, and pelvic tilt (PT) = 11° [19,20].

The reconstructed model was meshed using IAFE-MESH 1.0 software (University of Iowa, Iowa City, Iowa, USA and Hypermesh software 2016 (Altair, Troy, MI, USA). Linear hexahedral elements were used for modeling the cortical bone of the vertebrae and spinal discs. Linear tetrahedral elements were used for the cancellous bone of the vertebrae and the cortical and cancellous bones of the sacrum, pelvis, and femur. The ligamentous soft tissues were modeled using truss elements. The intervertebral disc included both the annulus and nucleus. The annulus and nucleus were simulated as a composite solid using hexahedral elements. The facet and sacroiliac joints were modeled using a surface-to-surface soft contact with exponential behavior.

The material properties were assigned based on previously published literature (Table 1) [21,22,23,24,25].

The model was previously validated by comparing the range of motion data against data obtained from studies on in-vitro cadaveric specimens. A mesh convergence study and validation of hip joint were performed on the model, as reported in a previous study [26]. Validation of lumbar spine and SIJ were performed and reported in a previous study [26].

### 2.2. Simulation of Pelvic Ring Fracture

**Figure 2:**
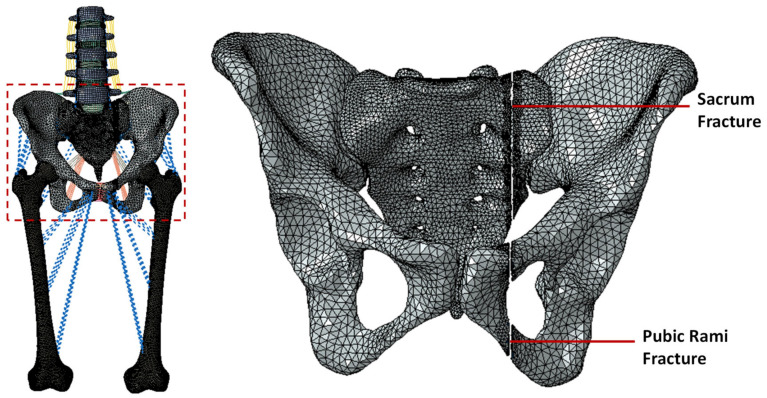
Simulation of the Rommens Type IIC pelvic ring fracture on the left side. The fracture was simulated by deleting the elements in the sacrum and ilium.

A unilateral pelvic ring fracture was simulated on the adult female spine FE model by resecting the left side of the sacrum and pelvis (deleting some elements of sacrum and pelvis) (Figure 2) [27]. A type IIC fracture was simulated as per Rommens classification [2] with a gap of approximately 1.7 mm. A surface-to-surface interaction with hard contact was used to model the interaction between the fractured surfaces of the sacrum and the pelvis [28].

### 2.3. Simulating the Instrumentation of Pelvic Ring Fracture

**Figure 3:**
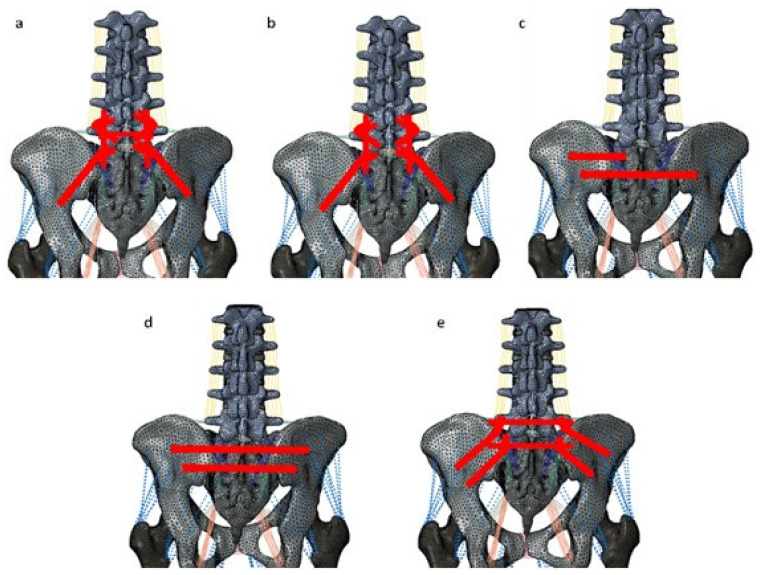
Stabilization of the pelvic ring fracture with various fracture fixation techniques. (**a**) indicates the L5-ilium fixation with cross connector (L5_PF_W_CC). (**b**) indicates the L5-ilium fixation without cross connector (L5_PF_WO_CC). (**c**) indicates the iliosacral screws at S1 and the trans-iliac–trans-sacral screw at S2 (S1_IS_S2_TITS). (**d**) indicates the trans-iliac–trans-sacral screw at both S1 and S2 (S1_TITS_S2_TITS). (**e**) indicates the double transiliac rod and screw fixation (DTSF).

This study simulated and evaluated five posterior instrumented stabilization strategies for pelvic ring fractures (Figure 3):

- L5-Ilium posterior screw fixation without cross connectors (L5_PF_WO_CC): Bilateral posterior screw fixation was performed from L5-Ilium. The pedicle screws were connected to spinal rods.
- L5-Ilium posterior screw fixation with cross connectors (L5_PF_W_CC): Bilateral posterior screw fixation was performed from L5-Ilium. The pedicle screws were connected to spinal rods. A cross connector was placed at the S1 level to connect the two rods.
- TITS fixation at S1 and S2 level (S1_TITS_S2_TITS): TITS fixation was simulated at the S1 and S2 levels.
- IS fixation at S1 and TITS fixation at S2 level (S1_IS_S2_TITS): A TITS fixation was simulated at the S2 level, and an ilio-sacral screw (IS) fixation was performed at the S1 level.
- Double transiliac rod and screw fixation (DTSF): Two traditional iliac screws were placed bilaterally. A horizontal rod was used to connect the iliac screws.

TiAl4V alloy material properties were applied to the screws, rods, and cross-connectors. A tie interaction was used to simulate the rigid fixation between the screws, the rods/screws, and the cross-connectors. A screw-bush and bush-bone interface was utilized to simulate the screw fixation. For the screw-bush interface, a tie interaction was defined between the outer surface of the screw and the inner surface of the bush. The bush-bone interface was simulated using the “coupling” constraint [29,30].

### 2.4. Loading and Boundary Conditions

For all models, a physiological loading of 400 N compressive follower load was applied to simulate the weight of the upper trunk and the effect of muscle forces [27]. A 7.5 Nm bending moment was applied to the superior surface of the L1 vertebra to simulate flexion/extension, lateral bending, and axial rotation [28]. The distal portion of the femur was fixed in all degrees of freedom to simulate a two-leg stance posture.

### 2.5. Data Analyses

The stabilization offered by the five models was quantified by analyzing the horizontal; and vertical displacement across the pelvic and sacral fractures. The standard protocol for sacral fracture fixation is typically the S1_IS_S2_TITS, therefore it was used to compare sacral fracture displacement across each case. The contralateral side (right) SIJ ROM, the L5-S1 ROM, L1-S1 ROM, and the L5-S1 nucleus stress were recorded and compared across the five instrumented models and to the intact model.

## 3. Results

### 3.1. Stabilization at the Sacral Fracture Region (Figure 4 and Figure 5)

At the fracture site, both horizontal and vertical displacement for S1_TITS_S2_TITS fixation showed the highest stabilization. At the same time, the L5_PF_WO_CC was noted to be the most unstable for horizontal displacement, however the DTSF was most unstable due to the large vertical displacement.

**Figure 4:**
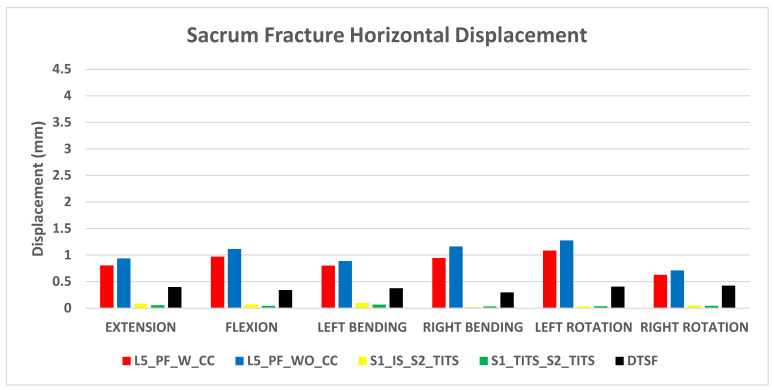
Comparison of sacrum fracture horizontal displacement at 7.5 Nm moment with 400 N follower load under two leg stance condition for five different pelvic ring fracture stabilization configurations. The vertical axis is the displacement in mm and the horizontal axis indicates the motions simulated.

Regarding the horizontal fracture site displacement, the L5_PF_W_CC configuration compared to S1_IS_S2_TITS showed more than 100% increases in extension, left bending, right bending, left rotation, and right rotation ROM, respectively. Similar trends were observed in L5_PF_WO_CC, and DTSF models, while S1_TITS_S2_TITS showed reduction in horizontal displacement at the fracture site compared to S1_IS_S2_TITS model. The reduction was approximately 32%, 34%, and 15% in extension/flexion, lateral bending, and axial rotation motions, respectively (Figure 4).

**Figure 5:**
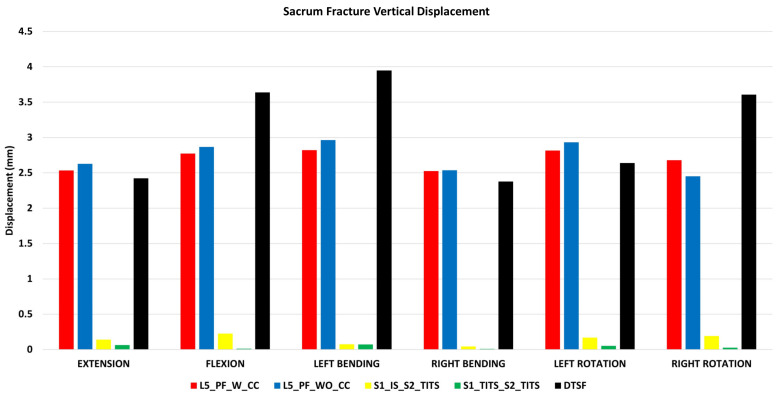
Comparison of sacrum fracture vertical displacement at 7.5 Nm moment with 400 N follower load under two leg stance condition for five different pelvic ring fracture stabilization configurations. The vertical axis is the displacement in mm and the horizontal axis indicates the motions simulated.

Regrading vertical fracture site displacement, L5_PF_W_CC, L5_PF_WO_CC and DTSF models compared to S1_IS_S2_TITS showed more than 100% increases for all motions. While S1_TITS_S2_TITS showed reduction in vertical displacement at the fracture site by approximately 55%, 94%,78%, and 86% in extension, flexion, right bending, and right rotation, respectively (Figure 5).

### 3.2. Stabilization at the Pubic Rami Fracture Region (Figure 6 and Figure 7)

The DTSF fixation showed the highest stabilization for horizontal and vertical displacement at the pubic rami fracture site. S1_IS_S2_TITS fixation resulted in largest horizontal displacement at the pubic rami fracture. The L5_PF_WO_CC fixation indicated the largest vertical displacement.

**Figure 6:**
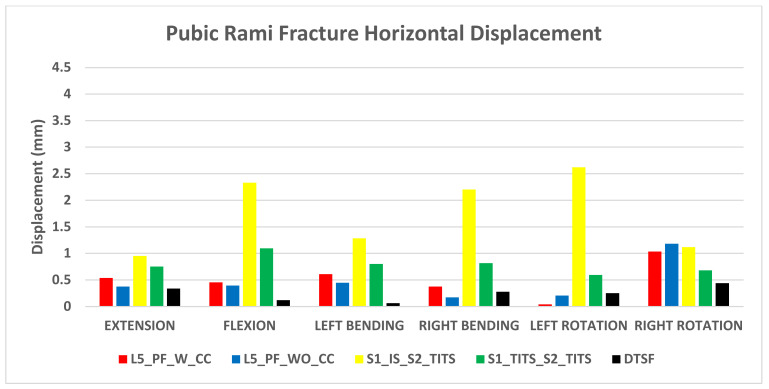
Comparison of Pubic Rami fracture horizontal displacement at 7.5 Nm moment with 400 N follower load under two leg stance condition for five different pelvic ring fracture stabilization configurations. The vertical axis is the displacement in mm and the horizontal axis indicates the motions simulated.

Regarding horizontal displacement, all other configurations showed approximately a 50% reduction in all motions compared to S1_IS_S2_TITS (Figure 6).

**Figure 7:**
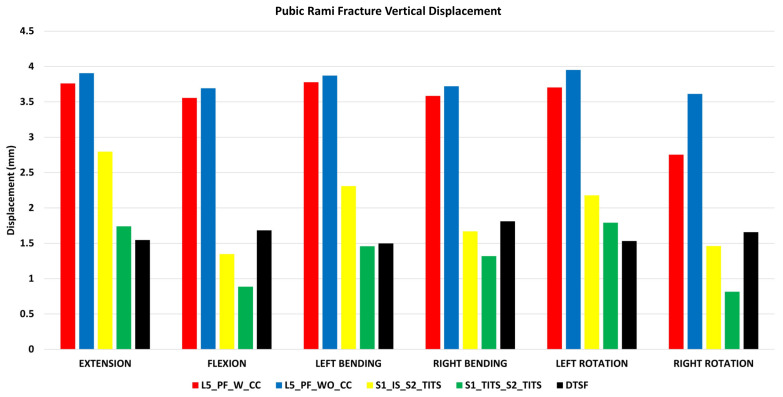
Comparison of Pubic Rami fracture vertical displacement at 7.5 Nm moment with 400 N follower load under two leg stance condition for five different pelvic ring fracture stabilization configurations. The vertical axis is the displacement in mm and the horizontal axis indicates the motions simulated.

For vertical displacement, the L5_PF_W_CC and L5_PF_WO_CC compared to S1_IS_S2_TITS showed increased displacement at pubic rami fracture site by 35%, 30%, 35%, 20%, 21%, and 40% for extension, flexion, left bending, right bending, left and right rotations, respectively. In contrast, the S1_TITS_S2_TITS and DTSF models showed decreased displacement at pubic rami fracture site compared to S1_IS_S2_TITS by approximately 35%, 165%, 65%, 120%, 75%, and 80% for extension, flexion, left bending, right bending, left and right rotations, respectively (Figure 7).

### 3.3. Sacroiliac Joint (SIJ) Range of Motion (ROM) (Figure 8)

The SIJ ROM ipsilateral to the fracture site (left SIJ) indicated little motion in all fracture fixation cases and therefore was not recorded. All SIJ ROM was compared to the intact model with no instrumentation. A reduction of SIJ ROM at the contralateral side (right side) was recorded with L5_PF_W_CC, L5_PF_WO_CC, and S1_TITS_S2_TITS configurations compared with the intact model. At the same time, S1_IS_S2_TITS showed increased ROM at the right SIJ compared with the intact model.

The S1_IS_S2_TITS fixation demonstrated the highest increase in the SIJ ROM at the contralateral side (right side) in flexion, extension, left bending, left axial rotation, and right axial rotation when compared to the intact model (121% 100% 164% 325% and 178%, respectively). Similar trends were observed with L5_PF_WO_CC compared to the intact model.

**Figure 8:**
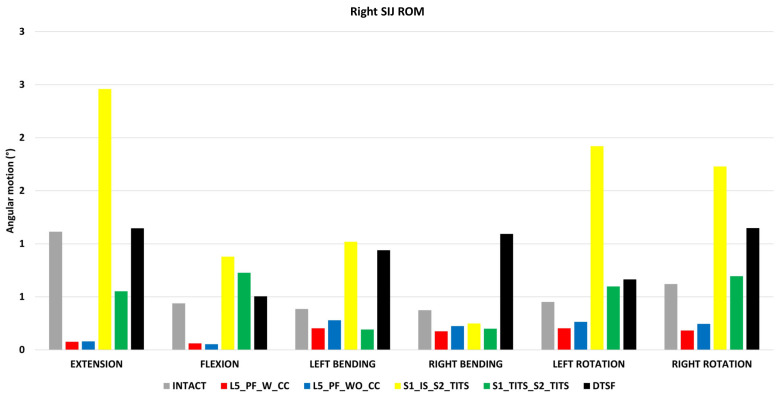
Comparison of right (contralateral to fracture site) SIJ ROM at 7.5 Nm moment with 400 N follower load under two leg stance condition for five different pelvic ring fracture stabilization configurations. The vertical axis is the angle in degrees and the horizontal axis indicates the motions simulated.

The L5_PF_W_CC fixation demonstrated the highest decrease in SIJ ROM at the contralateral side (right side) in flexion, extension, left bending, right bending, left axial rotation, and right axial rotation compared to the intact model (93%, 86%, 47%, 53%, 55% and 71%, respectively). Similar results were observed in the L5_PF_WO_CC model compared with the intact model (Figure 8).

### 3.4. ROM at the L5-S1 Level (Figure 9)

**Figure 9:**
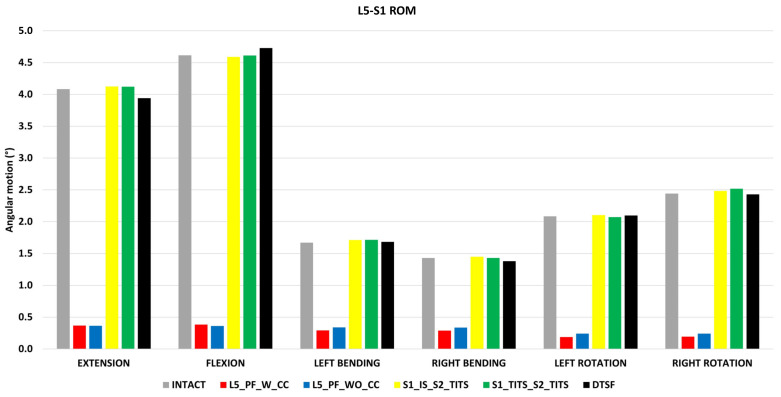
Comparison of L5-S1 ROM at 7.5 Nm moment with 400 N follower load under two leg stance condition for five different pelvic ring fracture stabilization configurations. The vertical axis is the angle in degrees and the horizontal axis indicates the motions simulated.

All cases for L5-S1 ROM were compared to the intact model with no instrumentation. The L5_PF_W_CC fixation showed the highest reduction in ROM at the L5-S1 level in flexion, extension, left lateral bending, right lateral bending, left axial rotation, and right axial rotation compared to the intact model (91%, 92%, 82%, 80%, 91%, and 92%, respectively). The L5_PF_WO_CC fixation also showed a substantial decrease in the ROM at the L5-S1 level. The ROM of the L5-S1 level showed negligible in the other stabilization models compared to the intact model (Figure 9).

### 3.5. Overall ROM for L1-S1 (Figure 10)

**Figure 10:**
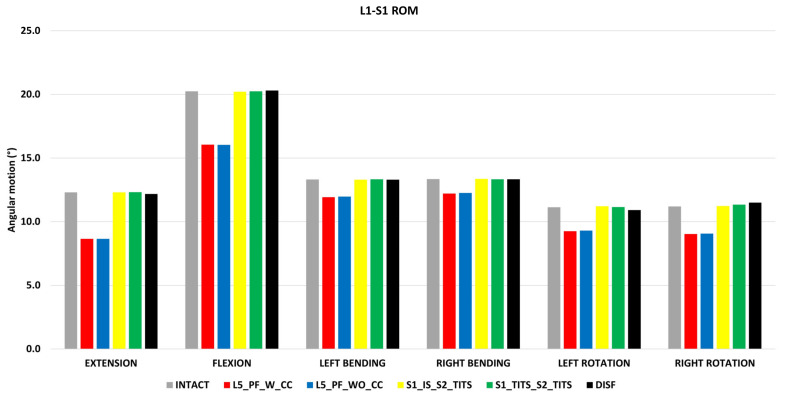
Comparison of L1-S1 ROM at 7.5 Nm moment with 400 N follower load under two leg stance condition for five different pelvic ring fracture stabilization configurations. The vertical axis is the angle in degrees and the horizontal axis indicates the motions simulated.

Compared to the intact model, L5_PF_W_CC and L5_PF_WO_CC indicated a reduction in ROM for L1-S1. The ROM was reduced by 29%, 20%, 10%, 8%, 16%, and 19% for extension, flexion, left bending, right bending, left rotation, and right rotation, respectively, in both configurations. There was no change in motion for other configurations at the L1-S1 level (Figure 10).

### 3.6. Peak von Mises Stresses at the L5-S1 Intervertebral Disc (Figure 11)

All surgical cases were compared to the intact model with no instrumentation. The L5_PF_W_CC showed the highest reduction in peak von Mises stresses in the L5-S1 nucleus (38%, 38%, 24%, 34%, 32%, and 20% decrease in flexion, extension, left and right lateral bending, and left and right axial rotation, respectively) compared to the intact model.

The L5_PF_WO_CC and S1_TITS_S2_TITS fixations also showed a substantial decrease in the von Mises stresses in the L5-S1 nucleus.

**Figure 11:**
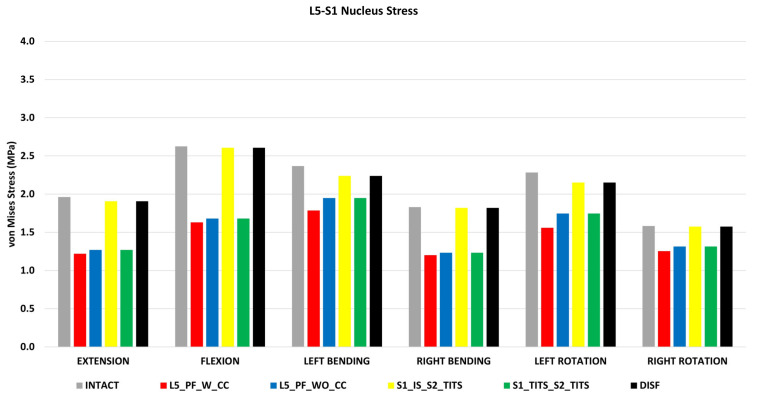
Comparison of L5-S1 Nucleus Stress at 7.5 Nm moment with 400 N follower load under two leg stance condition for five different pelvic ring fracture stabilization configurations. The vertical axis is the stress in MPa, and the horizontal axis indicates the motions simulated.

The von Mises stresses in the L5-S1 nucleus negligible changes for the other stabilization models compared to the intact model (Figure 11).

### 3.7. Stress on the Implants (Table 2)

Peak stresses on the rods and screws are shown in Table 2 for all the configurations for different loading conditions. Rods of L5_PF_WO_CC showed the largest stresses followed by L5_PF_W_CC and DTSF configuration. The screws of S1_TITS_S2_TITS indicated the higher stresses compared to the S1_IS_S2_TITS configuration.

**Table 2:** Maximum von Mises Stress (MPa) on the implants for each motion simulated.

Von Mises Stress (MPa)						
	Extension	Flexion	Left Bending	Right Bending	Left Rotation	Right Rotation
L5_PF_W_CC	310.2	251.7	368.8	209	279.3	266.8
	Left Rod	Left Rod	Left Rod	Left Rod	Left Rod	Left Rod
	Between L5 & ilium Tulip	Between L5 & ilium Tulip	Between L5 & ilium Tulip	Between L5 & ilium Tulip	Between L5 & ilium Tulip	Between L5 & ilium Tulip
L5_PF_WO_CC	308.2	287.9	403	212.4	307.6	307.3
	Left Rod	Left Rod	Left Rod	Left Rod	Left Rod	Left Rod
	Between L5 & ilium Tulip	Between L5 & ilium Tulip	Between L5 & ilium Tulip	Between L5 & ilium Tulip	Between L5 & ilium Tulip	Between L5 & ilium Tulip
S1_IS_S2_TITS	114.2	132.1	155.3	86.17	154.8	168.2
	TITS	TITS	TITS	TITS	TITS	TITS
S1_TITS_S2_TITS	194	188.9	243.6	200.3	188.1	218.2
	Top TITS	Top TITS	Top TITS	Top TITS	Top TITS	Top TITS
DTSF	75.76	81.97	71.3	114.4	94.15	99.15
	Top Rod	Top Rod	Top Rod	Top Rod	Top Rod	Top Rod

## 4. Discussion

To the author’s knowledge, this analysis is the first to evaluate the biomechanical effects of five different minimally invasive procedures for unstable pelvic fractures on the devices described, the fracture site and the lumbar spine.

During treatment of pelvic ring fractures, posterior stability is of paramount importance to ensure proper fracture union. Minimally invasive procedures have been proposed to avoid many of the complications associated with the traditional locking plate fixation used for sacral fractures; however, surgical treatment plans are highly dependent on the type of fracture pattern leading to debate on the optimal technique [31]. Due to this, we examined the biomechanical effects of various procedures including TITS, IS, DTSF and LP fixators on a Rommens Type IIC pelvic fracture.

In the present study, the cases that included the trans-sacral–trans-iliac screws (S1_IS_S2_TITS and S1_TITS_S2_TITS) resulted in superior stabilization of the sacral fracture compared to the other fixations. In an FE analysis for posterior internal fixation for unstable pelvic fracture, Zhang created fixation models with a single S1 screw (S1-1), single S2 screw (S2-1), two S1 screws (S1-2) and a combination of a single S1 and a single S2 screw (S1–S2). They concluded that for type C fractures, a two screw construct (S1–S2) allowed for biomechanical stability [32]. Our results in the present study display a similar outcome with the S1_IS_S2_TITS and S1_TITS_S2_TITS resulting in superior reduction of sacral fracture displacement compared to the other studied fixations, as indicated by the sustained sacral fracture stability in vertical displacement.

These results are consistent with Turbucz et al. who conducted a biomechanical study on a Denis Type II sacral fracture. They evaluated and compared the biomechanical efficacy of six iliosacral screw fixation techniques with different lengths on literature based and patient specific bone material properties. Their results indicated that the S1_TITS_S2_TITS had the highest vertical stabilization in the sacral fracture for all cases, including in osteoporotic bone [33]. It should be noted, however, that despite providing the highest stabilization in the sacral fracture region, the pubic rami fracture site showed the least stabilization in horizontal displacement in both fixation techniques. Furthermore, our study illustrated the sacral and pubic rami fractures were subject to less vertical displacement in the S1_TITS_S2_TITS compared to the S1_IS_S2_TITS, suggesting that inserting two longer trans-iliac–trans-sacral screws was more effective for fracture stabilization, agreeing with previous studies [34].

Moreover, S1_TITS_S2_TITS decreased contralateral SIJ ROM during extension and bending, compared to the intact model due to the longer screws fusing the joints.

S1_IS_S2_TITS produced increased contralateral SIJ ROM compared to the intact model due to the shorter IS screw not fusing the contralateral SIJ.

In both surgical cases that included SIJ fusion, negligible changes were seen in L1-S1 and L5-S1 ROM. Interestingly, there was a decrease in L5-S1 nucleus stress in the S1_TITS_S2_TITS, despite retaining L5-S1 ROM. This finding may be attributed to the higher sacral stability resulting from the fusion of both joints when using two trans-iliac–trans-sacral screws. These results agree with previous studies examining how SIJ fusion limits adjacent segment changes in the lumbar spine [24].

Regarding screw stresses, the fixation devices that assumed the greatest loading achieved the most fracture stabilization on the sacral region. When comparing the sacral fixation devices (S1_IS_S2_TITS and S1_TITS_S2_TITS), the S1_TITS_S2_TITS case indicated the highest implant stress and most stabilization on the sacral fracture compared to the S1_IS_S2_TITS case. Previous clinical results have noted high rates of screw loosening in IS and TITS fixations [35,36,37]. Our results numerically support these clinical findings as higher stresses on the screws may contribute to the risk of screw failure, especially when using two trans-iliac–trans-sacral screws. The higher screw stresses can be attributed to the TITS screws extending across and fusing both SIJs compared to the IS screw, which only fused the left SIJ. Bastian et al. further correlated screw breakaway within the sacrum and noted the superior portion of the sacrum provided optimal bone quality compared to the inferior portion for screw placement [35]. Moreover, the S2 segment is attributed by decreased bone density compared to the S1 segment [38] and has been clinically associated with S2 screw failures [39,40]. However, Eastman et al. revealed the region of the posterior ilium and SIJ to have the higher bone density compared to the sacral segments. This may indicate a decreased risk of screw failure for TITS placement due to crossing the posterior iliac region [41]. Interestingly, our results for the DTSF, placed in the posterior ilium, was also subject to the lowest stresses while reducing the pubic rami fracture more than the trans-iliac–trans-sacral fixations (S1_IS_S2_TITS and S1_TITS_S2_TITS). Clinical reports have noted trans-iliac screws have excellent results and lower complication rates compared to LCP and IS [8,13,42] which is further supported by the decrease implant stress noted by our study. Additionally, DTSF retained SIJ motion since the screws did not fuse the joint. L1-S1 and L5-S1 ROM are minimally changed compared to the intact which retains similar L5-S1 nucleus stress as the intact case. These outcomes warrant further investigation for DTSF as a favorable option for patients with poor sacral bone quality density. However, our results indicated that DTSF provided the least stabilization in the sacral fracture which may contribute to post-operative instability. Therefore, a delay in weight bearing conditions for patients treated with DTSF may be required to mitigate non-union at the cost of potentially unfavorable clinical outcomes [43].

Spinal instruments were originally applied with the Galveston technique for treatment of lumbosacral junction fractures. LP fixation with transpedicular screws are placed in the lower lumbar spine and additional screws in the posterior ilium [44]. Fixation in this regard is strong, however the mobility of the lumbopelvic region is restricted [45]. In this study, the LP fixations (L5_PF_WO_CC and L5_PF_W_CC) contributed to the greatest stability based on small horizontal displacement at the pubic rami fracture compared to the other studied fixations. The addition of a connector further increased the horizontal and vertical stability of the sacral fracture compared to the LP fixation without a cross connector. These results indicate certain fracture characteristics may benefit from cross connector due to increased facture site stability despite increased surgical time [46]. Thus, addition of a connector should be carefully considered depending on the instability of the fracture and other patient specific factors [47,48]. The fracture stability resulting from LP fixations allow for immediate weight bearing which poses an increased benefit for younger or certain at-risk patients [48]. However, based on our results, the stability of this construct comes into question as loosening of screws and instrument failure is possible as a result of the highest instrument stresses compared to the other fixation devices in this study. Additionally, L5_PF_WO_CC and L5_PF_W_CC led to the largest reduction of ROM and nucleus stress in the L5-S1 joint due to the fusion of the intervertebral levels. These fixations also produced the greatest decrease in the contralateral SIJ ROM, overall L1-S1 ROM and reduced the fused level’s nucleus stress. Previous clinical and biomechanical reports have suggested an increase in adjacent segment degeneration due to spinal fusions [49,50]. Our results may suggest a similar trend as stresses in other segments may increase due to the increased ROM at these regions Based on the highlighted limitations of LP fixations noted in our study, we suggest that these constructs should only be considered in patients with lumbosacral junction instability or patients with sacral instability not amenable to sacroiliac screw fixation [51]. However, further clinical studies are needed to support this theory.

### Limitations

This biomechanical study has some inherent limitations that need to be addressed. Firstly, absence of complex muscle forces, particularly to paraspinal muscles, was addressed by substituting them with follower load on the spine, while simplified elastic properties were employed for the lower extremity muscles. Secondly, the assumption was made that the implants and bones exhibit bonding. This research did not examine multiple screw diameters and lengths. However, previous biomechanical studies have confirmed that increasing the length or diameter of the iliac screws can significantly enhance the iliac fixation strength [52]. Different body weight and more osteoporosis were not simulated, and it will be interesting to explore these factors in future studies. This research relies on a limited number of finite element models, with only one model representing each configuration. This insufficient dataset poses a challenge for conducting statistical analysis, highlighting the need for additional patient models encompassing diverse races and ages. Nonetheless, it is believed that this limitation is unlikely to significantly impact the study’s findings.

## 5. Conclusions

The present study is the first to examine the mechanics of multiple fixation techniques for pelvic fractures and may assist clinicians in understanding their characteristics, advantages, and disadvantages.

## Figures and Tables

**Table 1:** Material properties assigned to the finite element model. E represents Young’s Modulus.

Component	Material Properties	Constitute Relation	Element Type
Vertebral Cortical Bone (17,554 nodes and 9123 elements)	E = 12,000 MPa	Isotropic, Elastic	8 Node Brick Element (C3D8)
	v = 0.3		
Vertebral Cancellous Bone (41,357 nodes and 202,515 elements)	E = 100 MPa	Isotropic, Elastic	4 Node Tetrahedral Element (C3D4)
	v = 0.2		
Pelvis Cortical Bone (Sacrum, Ilium) (26,490 nodes and 26,762 elements)	E = 17,000 MPa	Isotropic, Elastic	4 Node Tetrahedral Element (C3D4)
	v = 0.3		
Sacrum Cancellous Bone (16,891 nodes and 70,679 elements)	Heterogenous	Isotropic, Elastic	4 Node Tetrahedral Element (C3D4)
Ilium Cancellous Bone (20,083 nodes and 96,912 elements)	E = 70 MPa	Isotropic, Elastic	
	v = 0.2		4 Node Tetrahedral Element (C3D4)
Femur Cortical Bone (31,776 nodes and 31,776 elements)	E = 17,000 MPa	Isotropic, Elastic	4 Node Tetrahedral Element (C3D4)
	v = 0.29		
Femur Cancellous Bone (64,486 nodes and 334,529 elements)	E = 100 MPa	Isotropic, Elastic	4 Node Tetrahedral Element (C3D4)
	v = 0.2		
Ground Substance of Annulus Fibrosis (24,320 nodes and 18,480 elements)	c10 = 0.035		
	k1 = 0.296	Hyper elastic anisotropic (HGO)	8 Node Brick Element (C3D8)
	k2 = 65		
Nucleus Pulposus (14,326 nodes and 11,088 elements)	E = 1 MPa	Isotropic, Elastic	8 Node Brick Element (C3D8)
	v = 0.499		
Anterior Longitudinal (636 nodes and 553 elements)	7.8 MPa (<12%), 20 MPa (>12%)	Non-linear Hypo elastic	Truss Element (T3D2)
Posterior Longitudinal (636 nodes and 553 elements)	10 MPa (<11%), 20 MPa (>11%)	Non-linear Hypo elastic	Truss Element (T3D2)
Ligamentum Flavum (48 nodes and 24 elements)	15 MPa (<6.2%), 19.5 MPa (>6.2%)	Non-linear Hypo elastic	Truss Element (T3D2)
Intertransverse (66 nodes and 33 elements)	10 MPa (<18%), 58.7 MPa (>18%)	Non-linear Hypo elastic	Truss Element (T3D2)
Interspinous (60 nodes and 30 elements)	10 MPa (<14%), 11.6 MPa (>14%)	Non-linear Hypo elastic	Truss Element (T3D2)
Supraspinous (45 nodes and 23 elements)	8 MPa (<20%), 15 MPa (>20%)	Non-linear Hypo elastic	Truss Element (T3D2)
Capsular (45 nodes and 23 elements)	7.5 MPa (<25%), 32.9 MPa (>25%)	Non-linear Hypo elastic	Truss Element (T3D2)
Anterior SIJ (52 nodes and 26 elements)	125 MPa (5%), 325 MPa (>10%), 316 MPa (>15%)	Non-linear Hypo elastic	Truss Element (T3D2)
Short Posterior SI (20 nodes and 10 elements)	43 MPa (5%), 113 MPa (>10%), 110 MPa (>15%)	Non-linear Hypo elastic	Truss Element (T3D2)
Long Posterior SI (32 nodes and 16 elements)	150 MPa (5%), 391 MPa (>10%), 381 MPa (>15%)	Non-linear Hypo elastic	Truss Element (T3D2)
Interosseous (45 nodes and 23 elements)	40 MPa (5%), 105 MPa (>10%), 102 MPa (>15%)	Non-linear Hypo elastic	Truss Element (T3D2)
Sacrospinous (32 nodes and 16 elements)	304 MPa (5%), 792 MPa (>10%), 771 MPa (>15%)	Non-linear Hypo elastic	Truss Element (T3D2)
Sacrotuberous Ligament (58 nodes and 29 elements)	326 MPa (5%), 848 MPa (>10%), 826 MPa (>15%)	Non-linear Hypo elastic	Truss Element (T3D2)
Gluteus Maximus	k = 344 N/mm		Connector Element
Gluteus Medius	k = 779 N/mm		Connector Element
Gluteus Minimus	k = 660 N/mm		Connector Element
Psoas Major	k = 100 N/mm		Connector Element
Adductor Magnus	k = 257 N/mm		Connector Element
Adductor Longus	k = 134 N/mm		Connector Element
Adductor Brevis	k = 499 N/mm		Connector Element
Rods (Titanium)	E = 120,000 MPa	Isotropic, Elastic	Hexahedral Element
	v = 0.3		
Pedicle Screws (Titanium)	E = 120,000 MPa	Isotropic, Elastic	Hexahedral Element
	v = 0.3		

## Data Availability

The data presented in this study are available.

## Abbreviations

FE: Finite Element; SIJ: Sacroiliac Joint; TITS: Transiliac-trans sacral screw: IS Iliac screw; L5_PF_W_CC: L5-ilium posterior screw fixation with cross connector; L5_PF_WO_CC: L5-ilium posterior screw fixation without cross connector; S1_TITS_S2_TITS: TITS fixation at S1 and S2 level; S1_IS_S2_TITS: IS fixation at S1 and TITS at S2 level; DTSF: Double transiliac rod and screw fixation; ROM: Range of Motion; LL: Lumbar Lordosis; SS: Sacral Slope; PT: Pelvic Tilt; PI: Pelvic Incidence.
